# Improving survival in metastatic renal cell carcinoma (mRCC) patients: do elderly patients benefit from expanded targeted therapeutic options?

**DOI:** 10.1007/s00345-022-04110-3

**Published:** 2022-08-02

**Authors:** Hendrik Eggers, Christoph Schünemann, Viktor Grünwald, Linda Rudolph, Maria-Luisa Tiemann, Christoph Reuter, Merle Freya Anders-Meyn, Arnold Ganser, Philipp Ivanyi

**Affiliations:** 1 Department of Hematology, Hemostasis, Oncology and Stem Cell Transplantation, Hannover Medical School, Hannover, Germany; 2 Comprehensive Cancer Center Lower Saxony, Hannover, Germany; 3 Interdisciplinary Working Group Renal Cell Cancer of German Cancer Society (IAG-N), Berlin, Germany; 4 Clinic for Internal Medicine (Tumor Research) and Clinic for Urology, Interdisciplinary Genitourinary Oncology at the West-German Cancer Center, Essen University Hospital, Essen, Germany; 5 Department for Hematology, Hemostaseology, Oncology and Stem Cell Transplantation, Hannover Medical School, OE 6860, Carl-Neuberg Str. 1, Hannover, Germany

**Keywords:** Renal cell cancer, Cytokine, Tyrosine kinase inhibition, Metastases, Age, Geriatric patients

## Abstract

**Introduction:**

Treatment advances in metastatic renal cell carcinoma (mRCC) have improved overall survival (OS) in mRCC patients over the last two decades. This single center retrospective analysis assesses if the purported survival benefits are also applicable in elderly mRCC patients.

**Methods:**

401 patients with mRCC treated at Hannover Medical School from 01/2003–05/2016 were identified and evaluated by chart review. Treatment periods were defined as 01.01.2003–31.12.2009 (P1) and 01.01.2010–31.05.2016 (P2). Age groups were defined according to WHO classes (≤ 60 years: younger, > 60–75 years: elderly and > 75 years: old). Descriptive statistics, Kaplan–Meier analysis and logistic regression were performed.

**Results:**

Median OS improved from 35.1 months in P1 to 59.1 months in P2. Sub-division into the respective age groups revealed median survival of 38.1 (95%-CI: 28.6–47.6) months in younger patients, 42.9 (95%-CI: 29.5–56.3) months among elderly patients and 27.3 (95%-CI: 12.8–41.8) months among old patients. Risk reduction for death between periods was most evident among old patients (young: HR 0.71 (95%-CI: 0.45–1.13, *p* = 0.2); elderly: HR 0.62 (95%-CI: 0.40–0.97, *p* = 0.04); old: HR 0.43 (95%-CI: 0.18–1.05, *p* = 0.06)). Age ≥ 75 years was an independent risk factor for death in P1 but not in P2.

**Conclusion:**

Improved OS in the targeted treatment period was confirmed. Surprisingly elderly and old patients seem to profit the most form expansion of therapeutic armamentarium, within the TKI-dominated observation period.

**Supplementary Information:**

The online version contains supplementary material available at 10.1007/s00345-022-04110-3.

## Introduction

400.000 patients have been diagnosed worldwide with renal cell carcinoma (RCC) in 2018 [1]. Currently the mean age of RCC diagnosis in Germany is 70 years, and patients often exhibit significant comorbidity or fragility [2].

Twenty years ago, mRCC treatment options were limited to interferon and IL-2 conferring a median overall survival (OS) of approximately 13 months [3]. Collectively, Sunitinib and Sorafenib changed the medical treatment landscape, introducing the decade of the VEGF-R-Tyrosine kinase inhibition (TKI) [4]. Checkpoint inhibitors (CPI) have also entered the fray, with nivolumab being approved and demonstrating for the first time an OS benefit with second line treatments [5, 6]. Developing strategies further, CPI-doublet and CPI/TKI combination therapies have entered clinic practice, moving towards a new post-TKI-Mono-therapy era [7, 8].

It has, however, been well-documented, that both combined or sequential use of the aforementioned agents is accompanied by a variety of adverse events. This may be particularly pertinent in elderly patients, who not only may be particularly susceptible to chronic toxicity but also traditionally underrepresented in the relevant clinical trials. It remains largely unknown if older patients benefit in terms of OS from these targeted treatments, or improved numbers of therapeutic options. Data in other forms of cancer suggest that treatment advances do not necessarily improve OS in older sub-populations [9].

Patients ≥ 65 years represent only 30–40% of study populations in most pivotal phase 3 trials [10]. This is of particular relevance in RCC, given that the median age of diagnosis is around 70 years [2]. Therefore, here we evaluate if elderly patients demonstrate similar benefits in OS following the introduction of these various novel treatments, mainly with an observation period wherein therapeutic landscape was mainly enriched by TKI.

## Patients and methods

### Study design and data acquisition

All mRCC patients treated at our center (Department of Hematology, Hemostasis, Oncology and Stem Cell Transplantation, Hannover Medical School, Hannover) between 1 January 2003 until 31 May 2016 were identified. Patient and tumor characteristics, along with treatment data were evaluated by chart review. Inclusion criteria were: Age > 18 years, date of diagnosis of mRCC, application of first line treatment with targeted therapy, documented adherence to respective treatment guidelines and local standards of care.

Data were anonymized prior to storage and assessmentwas done by physicians and data managers in accordance to the both recommendations of the local ethics committee (No: 3171-2016) as well as the declaration of Helsinki in its latest version. Last follow up was performed on 31.05.2016. Data were stored in a Microsoft Access database. After documentation plausibility control was performed.

### Statistical analyses

Patients were divided into two time-dependent subgroups for comparison: those commencing treatment between 01.01.2003 and 31.12.2009 (period 1) and those starting between 01.01.2010 and 31.05.2016 (period 2). Period cut-offs were arbitrarily chosen to generate two samples of comparable size, but also reflecting to some extend the market authorization of the first TKI, considering a slow transformation of real world treatment patterns. Herein, sequential treatment applications, being affected by introduction of targeted therapies, disabled a certain approval date of targeted therapy as selection cut off. Furthermore “extended access programs” and clinical trials in our center as well as a heterogeneous implementation of new therapies in Germany with patients being referred to our clinic only after first line therapy failure create a major limitation and bias to define a reliable cut off by market authorization. Age subgroups were defined according to WHO as young (≤ 60 years), older (60.1–75 years) and old (> 75 years) at start of first systemic therapy [11].

Group comparisons were performed using Chi-square test, Fisher’s exact test, and *t*-test as applicable. OS was calculated from first diagnosis of metastatic disease (i.e. mRCC) until death or last follow up. Patients lost in follow up were censored at time of last documented follow up. Kaplan–Meier analysis was applied for overall population and subgroups and compared using the log-rank test. Univariate Cox regression models were performed to evaluate potential OS risk factors. Multivariable analysis was only applied for variables with statistical significance in univariate analysis. Missing values meet missing-completely-at-random criteria, meaning there is no significant bias on cox regression analysis [12]. A two-sided *p*-value below 0.05 was considered as statistically significant. SPSS 21.0 was used for statistical analysis (IBM, Armonk, New York). Documented parameters in the Access Data base were controlled prior to SPSS import.

## Results

### Patient and treatment characteristics

In total, 314 /401 patients with mRCC treated at our institution in the observation period fulfilled the predefined inclusion criteria (Supplement Fig. 1). Patients were not included due to missing date of mRCC diagnosis, missing date of therapy initiation and because they never received systemic therapy. Age distribution in patients not receiving systemic therapy was similar to those who received systemic therapy with a median OS of 46.2 months (95%-CI 3.3–89.1 months). Out of 314 patients who received systemic therapy, 173 patients (55%) were treated in period 1 and 141 (45%) in period 2. Median follow-up in period 1 was 33.8 (range (*r*): 1–181.6) months, compared to 27.3 (*r*: 0.6–179.5) months in period 2.

**Fig. 1:**
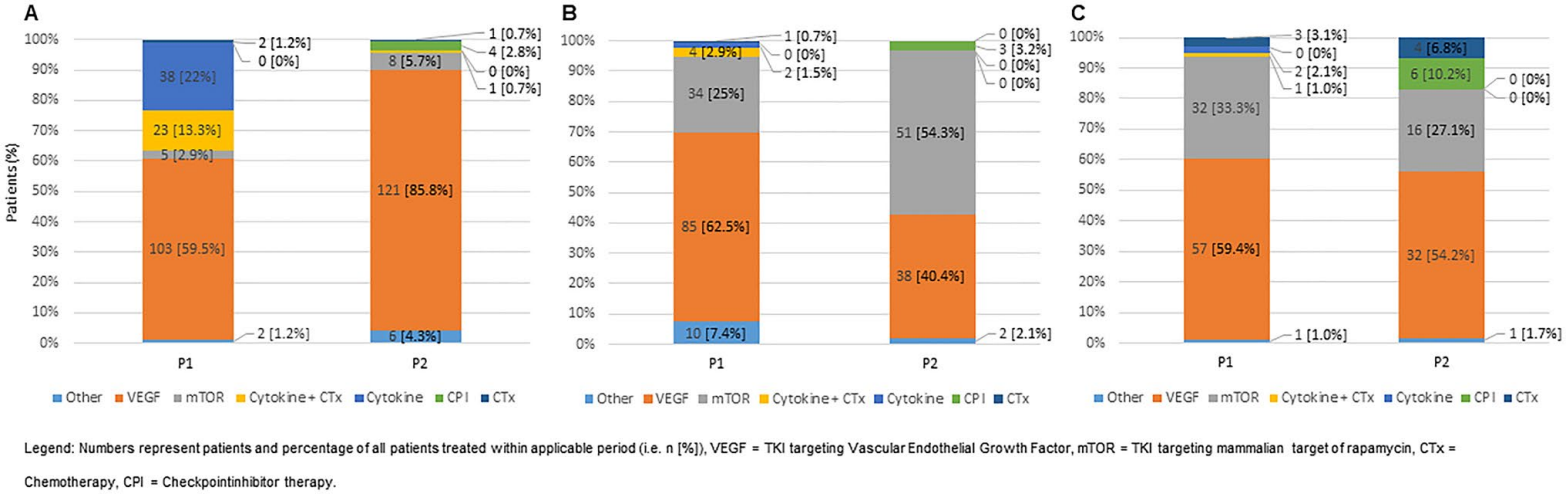
Treatment patterns in dependence of periods (P1, P2). **A** First line therapy by mechanism of action. **B** Second line therapy distribution by mechanism of action. **C** Third line therapy by mechanism of action

No significant differences in the majority of patient demographics between the two periods were observed (Table 1). However, patients in the second period tended to be older, although this didn’t achieve significance (*p* = 0.086). There were significantly higher rates of synchronous metastasis (42% vs. 29%, *p* = 0.015) and lung metastasis (59% vs. 48%, *p* = 0.043) in the earlier period, whereas performance status tended to be poorer in the more recent period (ECOG ≥ 1: 14 vs. 20 patients, *p* = 0.63). Conversely, these targeted period patients exhibited higher grade histology and received more complex medical strategies (grading: *p* = 0.001, therapeutic lines, *p* = 0.005) (Table 1). Inevitably, shifts in treatment strategies arose between periods, moving from cytokine based therapies towards TKI and mTOR inhibition were observed (Fig. 1). In first line therapy only 59.5% received TKI in period 1 compared to 85.8% in period 2, while cytokine-based therapies shrunk from 35.3% in period 1 to 2.8% in period 2 (Fig. 1).

**Table 1:** Patients characteristics

	Total, *N*	Period 1, *N* (%)	Period 2, *N* (%)	*p* value
Treatment period				
Total	314	173 (55)	141 (45)	
Gender				
Male	218	114 (66)	104 (74)	0.133
Female	96	59 (34)	37 (26)	
Age				
< 60 years	145	88 (51)	57 (40)	0.086
60.1–75 years	137	72 (42)	65 (46)	
> 75 years	32	13 (7)	19 (14)	
Histology				
Clear cell	243	139 (80)	104 (73)	0.529
Non clear cell	40	25 (15)	15 (11)	
Missing	31	9 (5)	22 (16)	
Grading				
G1/G2	161	107 (62)	54 (38)	0.001
G3/G4	99	46 (27)	53 (38)	
Missing	54	20 (11)	34 (24)	
Metastasis				
Synchronous	114	73 (42)	41 (29)	0.015
Metachronous	199	99 (57)	100 (71)	
Missing	1	1 (1)	0 (0)	
ECOG				
0	190	115 (67)	75 (53)	0.063
≥ 1	52	24 (14)	28 (20)	
Missing	72	34 (19)	38 (27)	
MSKCC				
Low	34	21 (12)	13 (9)	0.825
Intermediate/high	109	65 (38)	44 (31)	
Missing	171	87 (50)	84 (60)	
No. of organ systems				
0–1	152	83 (48)	69 (49)	0.866
≥ 2	162	90 (52)	72 (51)	
Pulmonary metastasis				
Yes	169	102 (59)	67 (48)	0.043
No	145	79 (41)	74 (52)	
Liver metastasis				
Yes	56	30 (17)	26 (18)	0.800
No	258	143 (83)	115 (82)	
Lympnodal metastasis				
Yes	107	62 (34)	45 (32)	0.466
No	207	111 (66)	96 (68)	
Soft tissue metastasis				
Yes	27	17 (10)	10 (7)	0.390
No	287	156 (90)	131 (93)	
Brain metastasis				
Yes	6	2 (1)	4 (3)	0.279
No	308	171 (99)	137 (97)	
Bone metastasis				
Yes	91	47 (27)	44 (31)	0.433
No	223	126 (73)	97 (69)	
Lines of medical treatment				
Mean (range)	2.74 (1–10)	3.02 (1–10)	2.38 (1–6)	< 0.001
Alive				
Yes	92	13 (8)	79 (56)	< 0.001
No	222	160 (92)	62 (44)

### Overall survival analysis

**Fig. 2:**
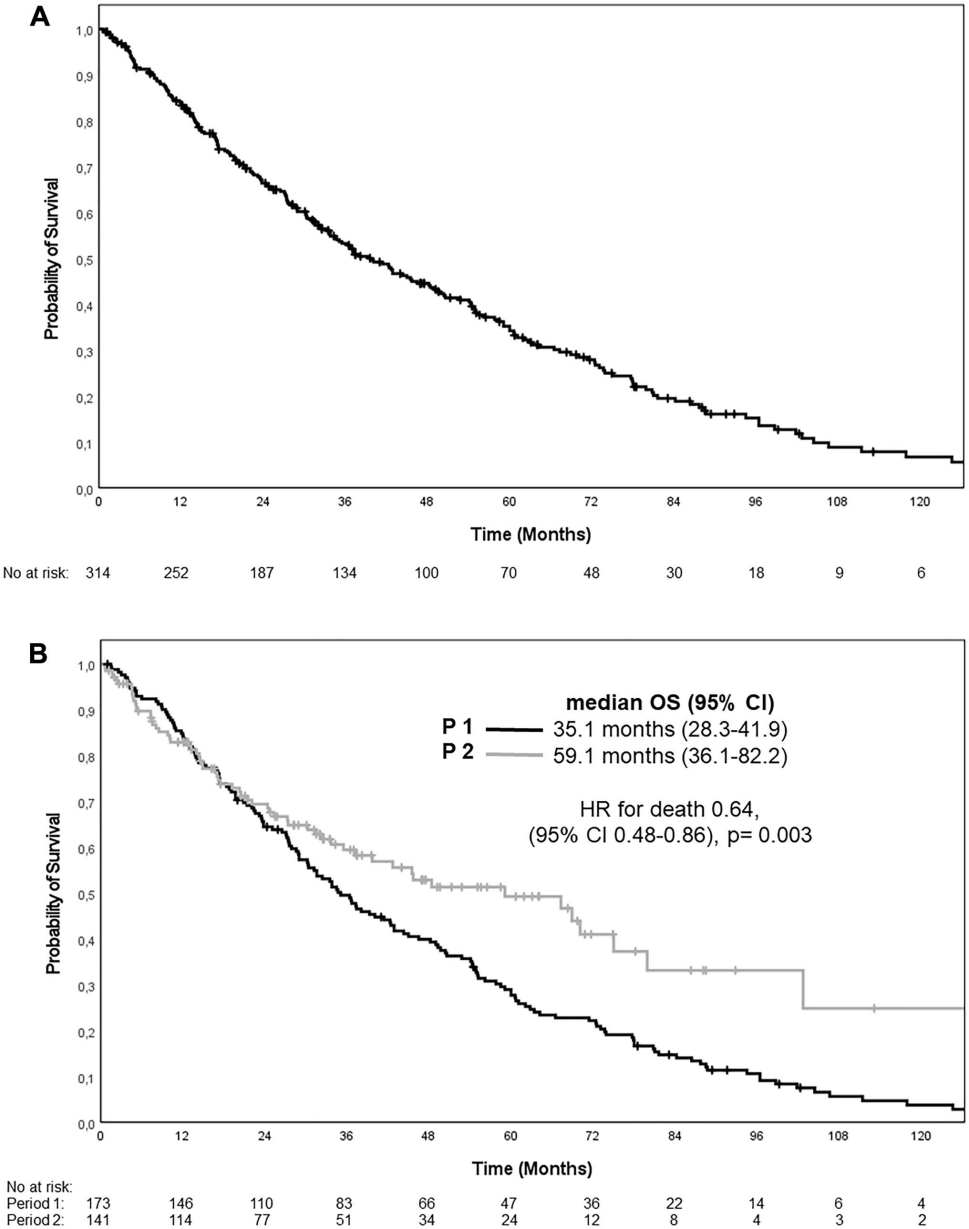
Overall survival in dependence of treatment periods (P1, P2). **A** Overall treatment period (2003–2016, *n* = 314 patients). **B** Treatment period 2 (2010–2016, *n* = 141) versus period 1 (2003–2009, *n* = 173)

Within the entire cohort, median OS was 39.7 (95%-CI: 33.1–46.3) months within the complete observation period (Fig. 2A), being much improved in the more recent compared to the earlier period (59.1 [95%-CI: 36.1–82.2] vs. 35.1 [95%-CI: 28.3–41.9] months; Log-rank: *p* = 0.002, Fig. 2B).

Within the defined age groups, OS among younger, elderly, and old patient groups was 38.1 (95%-CI: 28.6–47.6), 42.9 (95%-CI: 29.5–56.3) and 27.3 (95%-CI: 12.8–41.8) months, respectively. No significant inter-group differences in OS were observed.

**Fig. 3:**
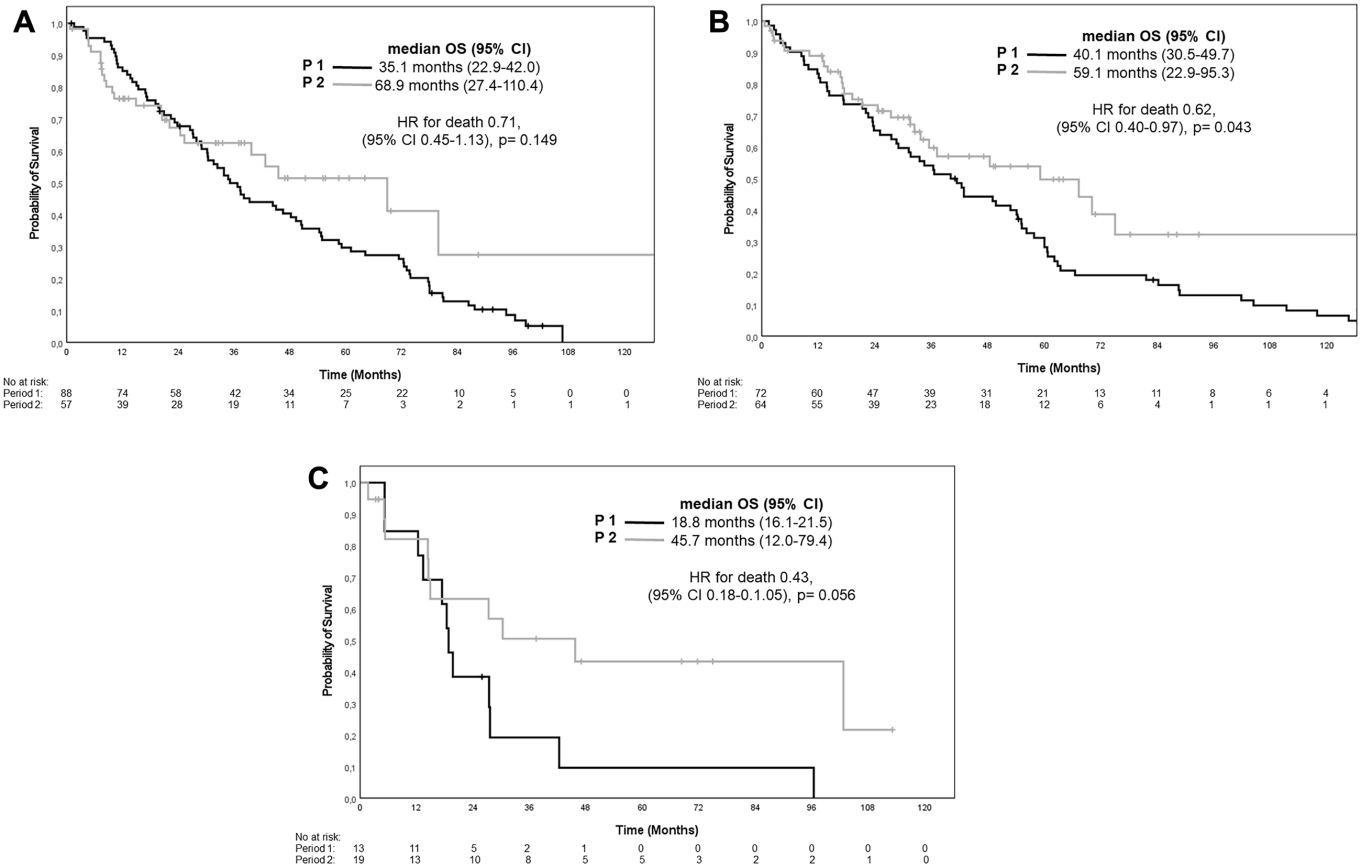
Overall survival in dependence of Age-group and treatment period 1 and 2. **A** Younger patients (≤ 60 years) period 2 vs. period 1. **B** Elderly patients (60.1–75 years) period 2 vs. period 1. **C** Older Patients (> 75 years) period 2 vs. period 1

Comparing OS within age-groups between treatment periods, no improvements were apparent in young patients (OS 68.9 (95%-CI: 27.4–110.4) months vs. 35.1 (95%-CI: 28.2–42.0) months; Log-rank *p* = 0.149; Fig. 3A). Early deaths in period 2 are probably causing a non-significant difference in OS of young patients, while in particular ECOG and MSKCC are equally distributed in this subgroup. By contrast, significant improvements among elderly patients (59.1 (95%-CI: 22.9–95.3) months vs. 40.1 (95%-CI: 30.5–49.7) months, Log-Rank *p* = 0.034; Fig. 3B) were observed, with similar trends being evident among old patients (45.7 (95%-CI: 12.0–79.4) vs. 18.8 (95%-CI: 16.1–21.5) months; Log-rank *p* = 0.056; Fig. 3C). Interestingly, the greatest reduction in risk of death was observed in old patients in period 2 vs. period 1 (Hazard ratio [HR] 0.43, 95%-CI: 0.18–1.05, *p* = 0.06, log rank, Fig. 3C).

### Risk factors for overall survival in dependence of different treatment periods

Several independent OS risk factors were identified. In the early period, multivariable analysis revealed age > 75 years (HR: 4.32, 95%-CI: 1.69–11.02, logistic regression: *p* = 0.002), non-ccRCC histology (HR: 2.79, 95%-CI: 1.48–5.27, logistic regression: *p* = 0.002), MSKCC (HR: 1.94, 95%-CI: 1.16–3.26, logistic regression: *p* = 0.012) and ≥ 2 sites of metastatic involvement as independent risk factors for OS (HR: 2.03, 95%-CI: 1.13–3.66, *p* = 0.018, logistic regression) (Table 2). In the more recent period, only ECOG ≥ 1 performance score was found to influence OS (HR 7.04, 95%-CI: 3.79–13.10, logistic regression: p < 0.001). Specifically, in Period 2 in contrast to period 1 age could not be identified as increasing risk in OS (Table 2).

**Table 2:** Risk factor analysis for death

Prognostic factor	Survival							
	Period 1				Period 2			
	Univariate		Multivariate		Univariate		Multivariate	
	HR (95% CI)	*p-value*	HR (95% CI)	*p-value*	HR (95% CI)	*p-value*	HR (95% CI)	*p-value*
*Gender*								
Male	1				1			
Female	1.01 (0.73–1.41)	0.937	-	-	1.60 (0.93–2.76)	0.089	-	-
*Age*								
< 60 years	1				1			
> 60 years	0.98 (0.71–1.34)	0.889	-		0.94 (0.56–1.57)	0.937	-	
< 75 years	1		1		1			
> 75 years	**1.97 (1.08–3.57)**	**0.026**	**4.32 (1.69–11.02)**	**0.002**	1.15 (0.58–2.28)	0.691	-	
*Histology*								
ccRCC	1		1		1			
Non-ccRCC	**2.16 (1.38–3.36)**	**0.001**	**2.79 (1.48–5.27)**	**0.002**	1.95 (0.94–4.04)	0.074	-	
*Grading*								
G1 + G2	1		1		1			
G3 + G4	**1.98 ( 1.38–2.85)**	** < 0.001**	**1.94 (1.16–3.26)**	**0.012**	1.63 (0.91–2.90)	0.100	-	
*Metastasis*								
Synchronous	1				1		1	
Metachronous	0.78 (0.57–1.08)	0.133	-		**0.58 (0.34–0.98)**	**0.041**	0.70 (0.39—1.28)	0.246
*ECOG*								
0	1				1		1	
≥ 1	1.48 (0.93–2.38)	0.101	-		**7.25 (3.91–13.44)**	** < 0.001**	**7.04 (3.79—13.10)**	** < 0.001**
*MSKCC*								
Low	1				1			
Int./high	**1.87 (1.11–3.14)**	**0.018**	**2.03 (1.13–3.66)**	**0.018**	2.41 (0.92–6.28)	0.073	-	
*No. of Organ Systems*								
0–1	1		1		1		1	
≥ 2	**1.69 (1.23–2.32)**	**0.001**	**1.94 (1.15–3.29)**	**0.014**	**1.35 (1.06–1.72)**	**0.015**	1.34 (0.74–2.42)	0.337

Significance is provided by p-values within the correlating column are shown in bold

## Discussion

Medical treatment options for mRCC have changed dramatically in recent years, offering a variety of different effective treatments, enabling impressive improvements in OS [13]. The relevance of study outcomes in the typically older patients more commonly found in clinical practice has remained elusive, given their underrepresentation in clinical trials [10], especially given that in other forms of cancer it has been shown that elderly patients did not share reported OS benefits from such novel treatments [9, 14, 15].

This current retrospective analysis attempts to address this specific question, comparing survival outcomes dependent upon age at diagnosis of mRCC in different treatment periods. Herein, we focused on the landscape switch, resulting from introduction of mainly TKI and mTOR inhibitors, while the period of checkpoint inhibition (CPI) is hardly reflected due to the observation periods.

Within our entire cohort, substantial improvements in OS were apparent over time, improving to 59.1 months in period 2 compared to 35.1 months in period 1 (Log-rank *p* = 0.003). This improvement is particularly encouraging given that patients treated more recently exhibited higher tumor grades and poorer performance status. However, we observed lower rates of synchronous disease in period 2, which may have led to an overestimation of observed OS improvement. The implementation of CPI, due to the low frequency of administration (*n* = 13; within first to third line treatment) does not seem to introduce relevant bias. Acknowledging this improvement of OS the most relatable change within treatment pattern from period 1 to period 2 were a notable decline in utilization of cytokines in first line therapy and an increase in utilization of mTOR inhibition in second line therapy. None the less, as mentioned, our observation period allows no definite conclusion about CPI introduction to the therapeutic landscape.

Considering further age-related OS, over the entirety of the study no significant differences between groups were identified, with all age groups demonstrating improved OS in the targeted treatment period (young 35.1 vs. 68.9 months, elderly 40.1 vs. 59.1 months, old 18.8 vs. 45.7 months). In the young OS nearly doubled, but did not reach statistical significance probably due to early deaths that can unfortunately not be elucidated due to the retrospective character of this study. Surprisingly, there was a notable risk reduction for death in the targeted treatment period particularly in old patients (57%; young: 29%, elderly 38%). This would suggest that targeted treatment options, like TKI and mTOR-inhibitor might be particularly beneficial towards OS in older patients. Underlining this further, is the elimination of age as an independent risk factor for death in the more recent period. An explanation for this remains beyond the scope of this study, but it does at least support use of these novel treatments especially in old patients that traditionally did not fit in the “one size fits all” approach due to comorbidities or frailty [14, 15]. Considering other malignancies, evidence can be found that careful patient selection may improve outcomes, particularly for frail patients and that generalized application of aggressive treatments may not be universally beneficial, and may indeed be harmful [14, 15]. Although we do not have sufficient data regarding adverse events to provide meaningful analysis, issues around age-dependent toxicity profiling remain highly interesting. Comprehensive geriatric assessment/screening is not currently routinely performed, but should be considered in future, and might further improve benefits in the old [16].

Given the limited available data for treating older mRCC patients, our study adds relevant information for this common and somewhat vulnerable patient population [10]. Median age in pivotal mRCC trials centers around 59–62 years, while real world data suggests that more than half of mRCC patient are diagnosed at median 65 years [2, 15, 17]. A recent update on the efficacy of the Javelin-renal-101 trial showed no difference in median progress-free survival or OS in patients aged ≥ 75 vs 65–75 [18]. This supports the hypothesis, that decision-making regarding targeted treatments in mRCC patients should be independent of patient age. Although the majority of our cohort were not CPI treated, we would advocate against increasing age being an unfavorable predictor of survival, and it cannot be justified as argument for withholding either VEGFR or mTOR inhibitors as a minimum and potentially all novel treatment options. Previous studies examining mRCC outcomes in geriatric patients present an incomplete picture: Bellmunt et al. have previously concluded that elderly and old mRCC patients exhibit equivocal benefit from sunitinib with respect to PFS and OS [19]. Pooled analysis of phase 2 and 3 trials in mRCC encompassing 4736 patients examined the question if age represented an independent prognostic determinant of OS. Stratified according to three age groups—young (< 50 years), intermediate (50–70 years) and elderly (> 70 years)—no significant differences in OS were observed, returning at 20, 17.3 and 21 months, respectively [20]. However, in that analysis, patients above 70 years did experience more adverse events, irrespective of improvements in OS [20]. It has also been demonstrated that mRCC patients ineligible for clinical trials tend to experience inferior outcomes with a median OS of 12.5 months for ineligible vs. 28.4 months for eligible patients [21]. This might be explained by chronic and cumulative toxicity of targeted medical therapies reducing net therapeutic benefit, particularly in elderly subjects [10]. Our study stands in line with previous findings suggesting, that elderly and old mRCC patients might profit substantially from therapeutic advances in mRCC treatment.

Doubtless, our study is limited by its retrospective nature, selection bias and the small number of patients within age subgroups, especially old patients > 75 years. Selection bias favors a beneficial outcome in the elderly as no patients without treatment were included. In conjunction with its retrospective nature, a significant amount of information was missing - in particular the MSKCC - and may lead to a relevant bias, although our multivariable model counteracts this bias. Perhaps more pertinent, is that the analyzed periods fail to adequately depict the fundamental transition from cytokine based strategies into the VEGF-era, nor do they reflect the overwhelming rapid change of the current treatment armamentarium with little CPI therapies included. Nonetheless, our hypothesis that elderly should also participate and benefit from expanded and more potent therapies is robust and highly relevant for decision-making in current clinical practice. In well selected patients targeted therapy might lead to comparable outcomes regardless of age. Our findings contribute further to findings in previous publications and reemphasizes the need for further evaluation, ensuring that a relevant proportion of patients, namely the old and the elderly, are counselled appropriately.

## Supplementary Information

Below is the link to the electronic supplementary material.

Supplementary file1 (TIF 96 KB)
