# Supplementary figures and images for: Improving survival in metastatic renal cell carcinoma (mRCC) patients: do elderly patients benefit from expanded targeted therapeutic options?
Source: World J Urol. 2022 Aug 2;40(10):2489–97. doi: 10.1007/s00345-022-04110-3 (PMC9512722; doi:10.1007/s00345-022-04110-3)

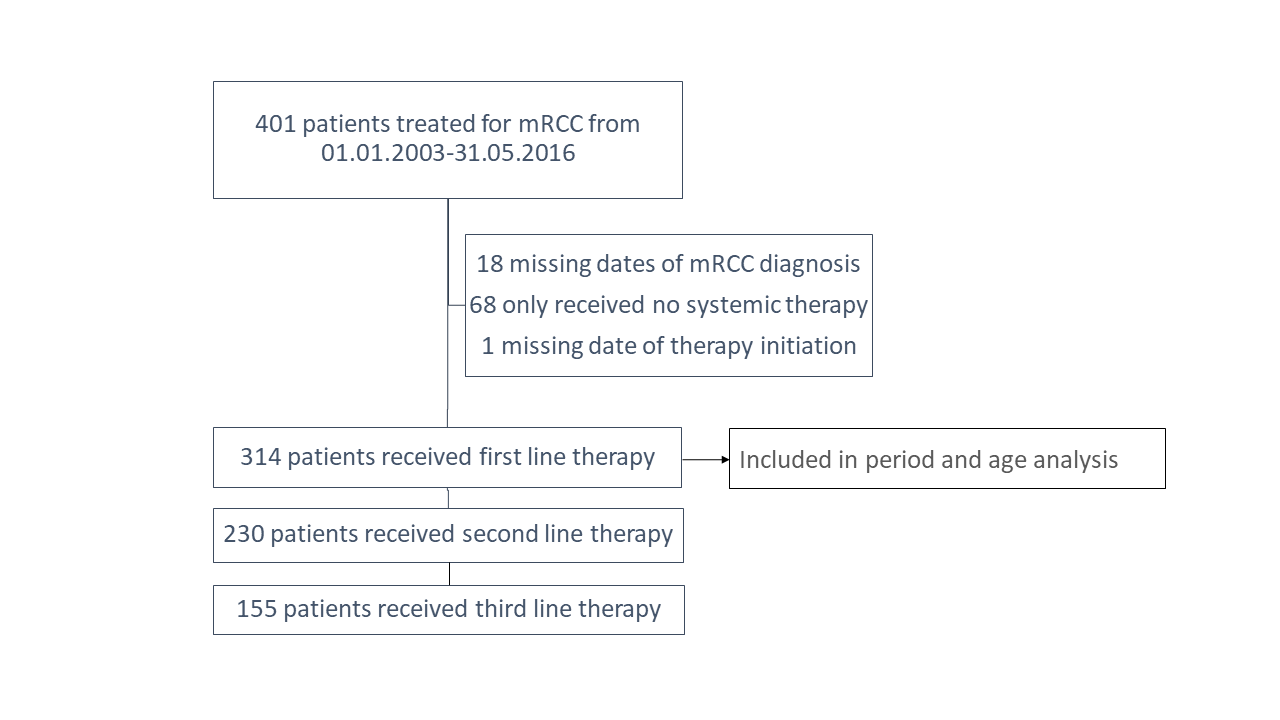

Supplement: Supplementary file 1 — Supplementary file1 (TIF 96 KB) [file 345_2022_4110_MOESM1_ESM.tif]
